# Effect of natural antioxidants on bond strength recovery of resin‐modified glass ionomers to the NaOCl‐affected pulp chamber dentin

**DOI:** 10.1002/cre2.697

**Published:** 2022-12-15

**Authors:** Fereshteh Shafiei, Zahra Dehghani, Maryam S. Tavangar

**Affiliations:** ^1^ Oral and Dental Disease Research Center, Department of Operative Dentistry, School of Dentistry Shiraz University of Medical Sciences Shiraz Iran; ^2^ Students' Research Committee Shiraz University of Medical Sciences Shiraz Iran; ^3^ Department of Operative Dentistry, School of Dentistry Shiraz University of Medical Sciences Shiraz Iran

**Keywords:** antioxidants, dental pulp cavity, sodium hypochlorite

## Abstract

**Objective:**

This study evaluated the effect of two natural antioxidants on the compromised bond strength of a resin‐modified glass ionomer (RMGI) to the sodium hypochlorite (NaOCl)‐affected pulp chamber dentin.

**Methods:**

Forty‐two sound third molars were split into halves. The exposed pulp chamber dentin was ground to provide the flat dentin surfaces and divided into seven groups (*n* = 12), according to the solutions used for immersion: (1) Control, distilled water; (2) NaOCl, 5.25% NaOCl for 20 min; (3) NaOCl/Ethylenediaminetetraacetic acid (EDTA); 5.25% NaOCl for 20 min + 17% EDTA for 1 min; (4) NaOCl/TA, 5.25% NaOCl + 10% tanic acid (TA) for 5 min; (5) NaOCl/EDTA/TA, 5.25% NaOCl + 17% EDTA + 10% TA for 5 min; (6) NaOCl/PA, 5.25% NaOCl+ 10% proanthocyanidin for 5 min; and (7) NaOCl/EDTA/PA, 5.25% NaOCl+ 17% EDTA + 10% PA for 5 min. The RMGI was bonded on the treated dentin using a Tygon tube. After 24 h of storage, microshear bond strength (µSBS) was tested. Data in MPa were submitted to one‐way analysis of variance and Tamhane test.

**Results:**

NaOCl significantly decreased the µSBS; NaOCl/EDTA and NaOCl/TA significantly increased the µSBS, higher than the control group (*p* < .05); and in the NaOCl/EDTA/TA group, the increased bond strength was at the level of the control group (*p* > .05). NaOCl/PA and NaOCl/EDTA/PA and NaOCl groups had comparable µSBS.

**Conclusion:**

TA could be suggested to provide effective bonding of RMGI and immediate sealing of the pulp chamber dentin after NaOCl irrigation.

## 1 INTRODUCTION

In addition to the apical seal, the establishment of an effective coronal seal immediately after endodontic treatment (ET) is a critical factor to achieve the final successful treatment (Nascimento Santos et al., 2006). Adhesively bonded restorations are well‐documented to provide a durable coronal seal (Fawzi et al., 2010). Furthermore, they are capable of strengthening the weakened tooth structure after ET and are applied as direct composite resin restoration or indirect composite/ceramic restorations, depending on the remaining tooth structure (Bohrer et al., 2018). Establishing effective bonding to the pulp chamber dentin is necessary for the initial step to prevent coronal microleakage (Bohrer et al., 2018). Resin‐modified glass ionomers (RMGIs) are considered an optimal material for immediate intraorifice sealing and can also be used as bases/liners in different direct/indirect adhesive restorations and core materials (Roberts et al., 2009). RMGIs have unique desirable properties and self‐adhesiveness, which provide an effective and durable bond to dentin. Also, their advantages are thermal compatibility with the tooth, sufficient working characteristics, and low water sensitivity (Atabek & Özden, 2019; Saad et al., 2017).

The pulp chamber dentin is a challenging bonding substrate because of variations in its structure. It contains a high density of enlarged tubules with low intertubular dentin, collagen‐rich predentin, and irregular secondary dentin (Fawzi et al., 2010). On the other hand, this structure is affected by different irrigants used in ET. Sodium hypochlorite (NaOCl) is the main irrigant used during instrumentation, and itself or in combination with ethylenediaminetetraacetic acid (EDTA) is applied for final irrigation. They could directly alter the interaction of the pulp chamber dentin with various adhesive materials. NaOCl decreases the mechanical properties of dentin and its microhardness. It leads to damage and partial removal of collagen fibrils, resulting in porous mineralized dentin; furthermore, it acts as a strong oxidizing agent, inhibiting resin polymerization (Pimentel Corrêa et al., 2016). Accordingly, applying an antioxidant agent might reverse this adverse effect (Farina et al., 2011). Sodium ascorbate was reported to restore the dentin bond strength of adhesive resin cement to the pulp chamber dentin, at least 50% of the original bond strength (Stevens, 2014).

Proanthocyanidin (PA) and tannic acid (TA) are natural polyphenols with antioxidant and collagen crosslinking properties that have been attractive in restorative dentistry (Cecchin et al., 2018). PAs have more potential to neutralize the oxidizing agents than that of sodium ascorbate, resulting in normal resin polymerization (Shi et al., 2003). The reversing effect of 6.5% PA on the compromised bonding of two adhesives to the NaOCl‐affected pulp chamber dentin has been reported (Chandrashekhar et al., 2018). This beneficial effect on the bond strength of a single bond was demonstrated for a 5‐min application of 10% PA and 10% TA solution at neutral pH (7.2) (Cecchin et al., 2018).

To date, this effect has not been assessed for RMGI, which is often directly applied to the pulp chamber dentin after ET is completed. The null hypothesis tested in this study was that applying PA and TA would not affect the bond strength of an RMGI, Fuji II LC (FLC), to the NaOCl or NaOCl/EDTA‐treated pulp chamber dentin.

## 2 MATERIALS AND METHODS

This in vitro study was conducted following approval by the Ethics committee for research (NO: IR.SUMS.DENTAL.REC 1399.052). Forty‐two freshly, caries‐free third molars were collected and stored in 0.5% chloramine T at 4°C until use. Each tooth was cut mesiodistally to expose the dentin surface of the pulp chamber. Each tooth incision was mounted in self‐curing acrylic resin, exposing the pulp chamber dentin. The dentin surface of the pulp chamber was ground with 600‐grit silicone paper under running water for 30 s before any treatments. All the materials used in the study are listed in Table 1.

**Table 1:** Materials utilized in the present study

Material	Manufacture	Batch number	Composition
FLC (Fuji ll LC)	GC Corp., Tokyo, Japan	180425C	Powder: Aluminosilicate glass Liquid: Water, polyacrylic acid, HEMA, UDMA, and camphorquinone
Tannic acid	Sigma‐Aldrich, USA	8186396	Tannic acid 10% weight, distilled water
EDTA	Morvabon, Iran	2118523	Ethylenediaminetetraacetic acid 17%
Proanthocyanidin	Puritanse Pride Inc, Oadale, NY, USA	768890	Proanthocyanidin‐grape seed extract 6.5% weight, distilled water

Abbreviations: EDTA, Ethylenediaminetetraacetic acid; FLC, Fuji II LC.

The 84 samples were then randomly divided into 7 groups of 12 each according to the different solutions used. (1) Control group: the samples were immersed in distilled water for 20 min; (2) NaOCl group: the samples were immersed in 5.25% NaOCl for 20 min and then washed with running water for 1 min; (3) NaOCl/EDTA group: after immersion in 5.25% NaOCl and washing, the samples were immersed in 17% EDTA (Sigma, Saint Louis, MO, USA) for 1 min and then washed with running water for 1 min; (4) NaOCl/TA group: after treatment with 5.25% NaOCl, the samples were immersed in 10% TA for 5 min; the solution was prepared using 10 gr of TA in the form of powder (Sigma, Saint Louis, MO, USA) dissolved in 100 ml of distilled water and then washed with water for 1 min; (5) NaOCl/EDTA/TA group: after treatment with NaOCl and EDTA, the samples were immersed in 10% TA for 5 min and then washed with water for 1 min; (6) NaOCl/PA group: after treatment with 5.25% NaOCl, the samples were immersed in 10% PA for 5 min; the solution was prepared using 10 gr of grape seed extract in the form of powder (Puritans Pride Inc., Oakdale, NY, USA) dissolved in 100 ml of distilled water and then washed for 1 min; and (7) NaOCl/EDTA/PA group: after preparation with NaOCl and EDTA, the samples were immersed in 10% PA for 5 min and then washed for 1 min.

After preparing the samples, RMGI (Fuji II LC, GC Corporation, Tokyo, Japan) was mixed according to the manufacturer's instructions and packed in cylindrical molds (Tygon tubes) with a diameter of 1 mm and a height of 1 mm, which was placed on dentin of the pulp chamber. The RMGI was then cured with a light‐curing unit (1200 mW/cm^2^, BlueLEX, Monitex) for 20 s. The bonded samples were kept in a humid environment for 24 h at 37°C. A wire of 0.2 mm diameter was inserted around the interface of the RMGI/dentin. The interface, wire loop, and load cell center were aligned as straight as possible. The microshear (µSBS) bond strength test was performed using a universal testing machine (Zwick‐Roell, Zwick, Ulm, Germany) at a speed of 0.5 mm/min. The debonded specimens were observed under a stereomicroscope at ×40. Fracture modes of the specimens were classified as follows: A: adhesive failure at the interface, B: cohesive failure in RMGI, and C: mixed failure, a combination of adhesive and cohesive failure. At first, the normal distribution of the data was analyzed using the Kolmogorov–Smirnov test and confirmed; then, the data were analyzed by one‐way analysis of variance and Tamhane post hoc statistical tests (*p* < .05).

## 3 RESULTS

The minimum, maximum, mean value, and standard deviation of the µSBS for the seven study groups are listed in Table 1. One‐way analysis of variance revealed a significant difference between the groups (*p* < .001). The Tamhane analysis test was applied to compare the groups. NaOCl significantly decreased the bond strength (*p* = .006). Compared to the control group, NaOCl/EDTA and NaOCl/TA treatment yielded a significantly higher bond strength (*p* = .03 and *p* < .001, respectively). However, the increased bond strength in NaOCl/EDTA/TA was not significant (*p* > .05). NaOCl/PA and NaOCl/EDTA/PA with similar results did not significantly affect the reduced bond strength of the NaOCl group (*p* > .05). Mixed failure was the main fracture mode in all groups, except for NaOCl, NaOCl/PA, and NaOCl/EDTA/PA groups in which adhesive failure was predominant (Table 2).

**Table 2:** Max, min, and mean (MPa) ± standard deviation of microshear bond strengths in the study groups (*n* = 12)

Group number	Group abbreviation	Mean ± SD	Min	Max	Fracture mode
					A/C/M
1	Control	8.69 ± 2.68^b^	5.2	12.5	3/0/9
2	NaOCl	3.98 ± 1.77^c^	1.8	6.5	8/0/8
3	NaOCl/EDTA	14.35 ± 3.88^a^	9.3	19.3	1/1/10
4	NaOCl/TA	15.89 ± 2.44^a^	11.7	18.7	0/3/9
5	NaOCl/EDTA/TA	11.59 ± 2.88^a,b^	8.3	16.8	0/2/10
6	NaOCl/PA	5.02 ± 2.86^c^	1.7	8.3	7/0/5
7	NaOCl/EDTA/PA	4.93 ± 2.36^c^	1.6	7.6	9/1/2

*Note*: The same superscript letters indicate no statistically significant difference between groups (*p* > .05).

Abbreviations: A, adhesive failure; C, cohesive failure; EDTA, Ethylenediaminetetraacetic acid; M, mixed failure.

## 4 DISCUSSION

In the current study, the pulp chamber dentin was treated with common irrigants with the same duration time applied in ET to simulate the clinical situation and RGMI (FLC) was bonded immediately. Accordingly, NaOCl led to a decrease in the µSBS of FLC. This negative effect has been previously reported for some adhesives to the pulp chamber dentin (Cecchin et al., 2018; Fawzi et al., 2010; Nascimento Santos et al., 2006; Stevens, 2014). Also, 1% NaOCl reduced the SBS of FLC to dentin (Sekhar et al., 2017). However, in another study, the reverse beneficial effect was demonstrated for Vitremer (Al‐Gerny et al., 2019). In two other studies, NaOCl was used as a disinfecting agent for 30 s (Al‐Gerny et al., 2019; Sekhar et al., 2017). The application time is a determinant factor in the NaOCl impact on adhesive bond strength (Sanon et al., 2022). Moreover, this effect is adhesive‐dependent. The oxidizing effect of NaOCl is more relevant for its usage for a relatively long time in ET (Coutinho et al., 2007).

In this regard, no study was reported on the interaction of pulp chamber dentin and RMGI with endodontic irrigants. The adverse effect reported in Sekhar and colleagues study was attributed to the dissolution of collagen, leading to the impedance of ionic bonding of RMGI to the dentin collagen and hybrid layer formation (Sekhar et al., 2017).

RMGIs are self‐bonded to the dentin structure through two mechanisms: ionic interaction between anions of polyalkenoic acid chains and calcium ions of hydroxyapatite around collagen and micromechanical interlocking, hybrid layer and resin tag formation into surface roughness and porosity resulted from the self‐etching ability of RMGI (Imbery et al., 2013; Yap et al., 2003).

The results of the current study demonstrated that applying EDTA, TA, and EDTA followed by TA could remarkably improve the SBS of FLC. According to previous studies, EDTA increased the bonding effectiveness of RMGI to the smear layer‐covered dentin (Fagundes et al., 2009; Saad et al., 2017). Despite the removal of the smear layer and smear plug by EDTA, mineral contents of intrafibrillar collagens were maintained to allow the chemical bonding to occur (Habelitz et al., 2002; Saad et al., 2017). NaOCl can dissolve the organic component of the smear layer (Fawzi et al., 2010); the dissolve collagens might interfere with the self‐adhesiveness of RMGI. Consequently, EDTA and TA could remove this degenerated layer, allowing calcium ions to bond with an ionic component of RMGI. TA contains carboxylic acid groups with weak acidity (Bedran‐Russo et al., 2009). It is capable of mild demineralizing and partially removing the smear layer (Christopher et al., 2016). Also, EDTA with a pH of 7.4 has these capabilities through chemical chelation, not acidity (Fawzi et al., 2010; Hülsmann et al., 2003). In contrast to TA, PA used after NaOCl was not able to restore the µSBS of FLC. Although the prepared PA solution had a slight acidity, its pH was adjusted to 7.2 by adding NaOH to the solution. These findings revealed that the antioxidant activity of PA and TA may have no benefit in the bonding reversal of RMGI to the NaOCl‐treated dentin. It seems that the other properties of PA and TA may contribute to the opposite results for TA versus PA. The beneficial effect of EDTA on the NaOCl‐affected pulp chamber dentin was reversed by PA application in the NaOCl/EDTA/PA group. PA with a large molecular size and several hydroxyl groups creates a strong bonding to proteins amid the carbonyl group of the collagen fibers via hydrogen ionic, covalent, and hydrophobic interactions (Hass et al., 2016). Furthermore, the hydroxyl groups of PA can create chemical bonding with calcium ions of dentin. Hence, these interactions may lead to interference with the micromechanical and chemical bonding of RMGI.

The two bonding mechanisms of RMGI could contribute to the bond strength of RMGI. However, its chemical bonding is essential to provide bonding durability (Saad et al., 2017). The microspecimens of the FLC‐pulp chamber dentin used in this bond strength study could provide suitably bonded microspecimens because they need no further preparation after the initial bonding procedures. This prevents the induction of prestress on the bonding interface before testing.

The null hypothesis tested in this in vitro study has to be partially rejected since TA has been revealed to exert a considerably beneficial effect on the bonding ability of the RMGI to the pulp chamber dentin, whereas PA had no effect.

The present study was performed on the smear layer‐covered dentin produced by grinding. This grinding is necessary to provide a flat dentin surface for bond strength testing. However, in the intraoral situation, RMGI was bonded directly on the pulp chamber dentin with no dominant smear layer because it is not prepared during ET (Schellenberg et al., 1992).

The present research was a short‐term bond strength study. Further long‐term research with different antioxidant/irrigant agents and various application times is suggested to be conducted to evaluate the longevity of the bonding of RMGIs.

In the current vitro study, we have only evaluated the short‐term effect of the two antioxidants on the bond strength of RMGI to NaOCl‐affected dentin. As the aging process may influence the durability of the bond, long‐term investigations with other antioxidant agents by using different application times are suggested. Furthermore, in vivo studies should be conducted before the advisement of antioxidants in clinical procedures.

## 5 CONCLUSION

The adverse effect of NaOCl irrigation on the bonding ability of the RMGI to the pulp chamber dentin could be completely recovered by a 5‐min application of TA solution. PA was not capable of inducing any reversing effect on the compromised bond strength.

## AUTHOR CONTRIBUTIONS

All authors contributed to the conception, design, data acquisition and interpretation, and statistical analysis and drafted and critically revised the manuscript. All authors gave their final approval and agree to be accountable for all aspects of the work.

## CONFLICT OF INTEREST

The authors declare no conflict of interest.

## Data Availability

The data that support the findings of this study are available on request from the corresponding author. The data are not publicly available due to privacy or ethical restrictions.
